# 

**Published:** 1918-11-09

**Authors:** 


### The Royal Sanitary Institute.

### RESULTS OF EXAMINATIONS.

THE examinations of the Royal Sanitary Institute were held at Liverpool on October 18 and 19. The following were awarded certificates:—

*Maternity and Child Welfare.*—Harris, Theodora (Derby); Hindle, Edith (Barrow-in-Furness); Sleigh, Florence (Rudyard).

*Inspectors of Nuisances.*—The following four candidates were certified, as regards their sanitary knowledge, competent to discharge the duties of inspector of nuisances under the Public Health Act, 1875: Aspden, Margaret (Bootle); Hodgson, James (Wallasey); Newell, Lily Pearce (Wallasey); Thomas, Daniel (Wrexham).

*Women Health Visitors and School Nurses.*—Five candidates presented themselves, but no certificates were awarded.

### Passing Events and Topics.

### Consumptive Ex-Service Men.

Lord Cheylesmore, the chairman of the committee of Brompton Hospital, appeals for gifts of clothing, especially overcoats, suits, boots, underclothing, and dressing-gowns, for the use of sailors and soldiers under treatment at the hospital who have been invalided out of the Services.

### The British Fire Prevention Committee,

The British Fire Prevention Committee, 8 Waterloo Place, Pall Mall, has issued a reprint of its useful fire precautions for householders, a copy of which may be obtained on application. At this time especially, when the number of resident male employees in institutions has been reduced to a minimum and help in the event of an outbreak of fire is not so readily forthcoming, all means of fire prevention should be carefully studied by those responsible for the safety of institutions.

### Great Northern Central Hospital.

Owing to the prevailing epidemic, the Ladies' Association of the Great Northern Central Hospital has decided to postpone the Annual Pound Day, which was fixed for November 14. The date selected will be notified as soon as circumstances permit.

### Matrons' and Sisters' Appointments.

General Hospital, Nottingham.—Miss May Stephens has been appointed massage sister. She was trained at the Royal Salop Infirmary, Shrewsbury, where she was later holiday sister. She holds the certificate of the Incorporated Society of Trained Masseuses.

Royal Hospital for Sick Children, Aberdeen.—Miss Ella Menzies has been appointed surgical sister. She was trained at the Edinburgh Royal Infirmary, where she has since been sister, and she has been sister-in-charge of Burcote Private Hospital, Berkshire.

Royal National Hospital for Consumption, Ventnor.—Miss Mary Cathrine Jones has been appointed sister. She was trained at Mount Vernon Hospital, Northwood, and at the Royal Berkshire Hospital, Reading; has been sister in the military wards at the last-named hospital, and sister on the private staff of the Royal Sussex County Hospital, Brighton.

Stockport Union Infirmary.—Miss Annie Maude Shepherd has been appointed night superintendent. She was trained at the Stepping Hill Hospital, Stockport, and has since been ward sister at the Union Infirmary, Rochdale, and night superintendent at Keighley Union Infirmary. She holds the certificate of the Central Midwives Board, and is a member of the College of Nursing.

Tehidy Sanatorium, Cornwall.—Miss Effie Bamford has been appointed matron. She was trained at the Mill Road Infirmary, Liverpool; has been matron of the Newcastle County Nose and Throat Hospital, Nordrach-on-Mendip Sanatorium, Wakefield Sanatorium, and the Middleton-in-Wharfedale Sanatorium.

**Note.—Particulars of Appointments for insertion in this column will be welcomed.**

### Proposed Nurses' League at Fulham Infirmary.

[Communicated.]

The nurses trained at the Fulham Infirmary met in the Nurses' Home at four o'clock in the afternoon of October 30 to take steps to form a League amongst themselves.

These came: Miss S. Shorrocks; Sisters L. A. Wallace, A. K. Andersson, M. Owen, S. M. Edwards, M. Croot, E. Hogan, J. Walsh, E. Harse, E. Bevan; Staff Nurses D. Page, Mary Kennedy, Margaret Kennedy, Mrs. A. Connors (who was Nurse A. Collins), and Mrs. M. J. Rylance (who was Nurse M. J. Atkinson).

These also came: The Matron, Miss J. F. Ballantyne; the Medical Superintendent, Dr. C. T. Parsons; the Chaplain and his wife, the Rev. R. V. Tremills and Mrs. R. V. Tremills; the Assistant Matron, Sister S. S. Bevan; Sisters L. Watts, A. Slarks, M. Brownsell, Mrs. M. Greet (who was Sister M. Harrison), Mrs. B. M. Ball (who was Nurse Smith), Mrs. G. Tarbet (who was Pro. Nurse Kingsley).

These wrote they would but could not come: Sister J. Comerford (Taormina, Sicily), Mrs. Giles (who was Sister A. Wright), Mrs. Gibbs (who was Sister A. Mason), Mrs. Millard (who was Sister Fail), Sister M. Roberts, Mrs. MacGregor (who was Nurse Nightingale), Nurses L. Toft, A. Davies, D. Butt, L. Balloch, and M. Haines.

The meeting, none gainsaying, agreed to form a League. Further, after much interchange of arguments, it fell of accord upon the matters that follow: (1) All nurses holding the training certificate of the infirmary to be members of the League upon payment of the fee of membership. (2) Strugs, Worfuns, and others closely linked with the school, after the publishment of their names, to be chosen to the League by the members. (3) The fee of membership to be five shillings for one year. (4) A meeting to be held at the Nurses' Home on Wednesday, December 11, at four o'clock in the afternoon. The things to be done by the meeting will be (*a*) the framing of the hidden and open rules of the League; (*b*) the choosing of a name, of the officers, and of a committee; (*c*) the opening of the Book of Words, of the Book of Deeds, and of the Book of Ceremonies. (5) These were chosen to frame the rules and to bring forward such matters as might seem to them to be for the good of the League: Miss Ballantyne, Dr. C. T. Parsons, Miss S. Shorrocks, Sisters Bevan, Wallace, Hogan, and Harse.

### The College of Nursing
### (Limited by Guarantee).

NEW LONDON HEADQUARTERS.

The new Headquarters of the College are to be in Henrietta Street, Cavendish Square, not very far from the present offices. It is probable that the London Centre of the College will rent one floor of the house for its own use, and the members of the Centre, who are keenly enthusiastic about having their own club, hope at an early date to make a united effort to raise the funds required for its furnishing and equipment.

BIRMINGHAM CENTRE. (Hon. Secretary, Elizabeth F. Colburn.)

Friday, November 15, 3 p.m.—Meeting at the General Hospital, Birmingham. Miss Gertrude Cowlin, Organising Secretary of the College of Nursing, Ltd., will speak on the aims and objects of the College. All nurses cordially welcomed.

YORKSHIRE CENTRE. (Hon. Secretaries, Miss Lindall and Miss Spann.)

A series of Post-Graduate Lectures, to be held at the Leeds General Infirmary, has been arranged which are free to members, and open to non-members for a fee of 2s. 6d. for the series, or 1s. each lecture. Tickets for the series and the syllabus can be obtained from the Hon. Secretaries, Miss Lindall, Hospital for Women and Children, Leeds, or Miss Spann, The Township Infirmary, Beckett Street, Leeds. The next lecture will be given on Thursday, November 21, by Dr. Clara Stewart, M.B., on "Venereal Diseases."

LEEDS CENTRE: HULL BRANCH.
(Hon. Secretary, Miss Dora Lyon.)

The following post-graduate lectures will be given at the Hull Royal Infirmary during the winter months at 8 p.m.: November 7: "Modern Anæsthesia," Dr. Florence Stacey. December 5: "My Experiences in Serbia," Miss Pritchard. January 9, 1919: Social Evening. February 6: "Wanderers of the Earth," Miss E. M. L. Elliott, M.A. March 6: "War Surgery," Major Coupland. The lectures are free to all trained nurses in Hull and district. Two lectures have already been given, one in October by Miss C. T. Cumberbirch, B.A., and one on the 7th inst. by Dr. Florence Stacey on "Modern Anæsthesia."

NORTHUMBERLAND AND DURHAM CENTRE.

At a meeting of members at the Royal Victoria Infirmary, Newcastle, on Tuesday, November 12, at 7.30 p.m., the Organising Secretary, Miss Cowlin, will speak upon the College of Nursing, Ltd., and the value of local Centres.

Miss Cowlin will also address meetings at the Royal Infirmary, Sunderland, on November 13, at four o'clock, and at St. William's College Hall, York, on November 14, at 3 p.m.

### Order the Paper.

In consequence of the No Returns Order of the Government, readers of The Hospital are requested to ensure a regular supply of the paper by placing an order with their newsagent or bookstall without fail.

HOSPITAL SATURDAY.

The following contributions are amongst those received at the head office in response to the annual appeal issued on Hospital Saturday, October 12, 1918:—Matinée at the London Palladium (Mr. Chas. Gulliver), £314 10s.; proprietors of the *Daily Telegraph*, £100; G. N. Railway Co. (Metropolitan stations), £81 1s. 2d.; L. and S. W. Railway Co. (Waterloo Station), £61 2s.; Covent Garden Market (Mr. S. Emanuel), £30 5s. 8d.; Frascati and Holborn Restaurants, £25 15s. 6d.; Aerated Bread Co., £25; and L. B. and S. C. Railway Co. (Victoria and London Bridge Stations), £21 0s. 5d.

